# Supplementary material for: Foliar Application of Silicon in Vitis vinifera: Targeted Metabolomics Analysis as a Tool to Investigate the Chemical Variations in Berries of Four Grapevine Cultivars
Source: Plants (Basel). 2022 Nov 7;11(21):2998. doi: 10.3390/plants11212998 (PMC9654429; doi:10.3390/plants11212998)
Supplement: Supplementary file 1 [file plants-11-02998-s001.zip › plants-1969184-supplementary.pdf]

Foliar Application of Silicon in Vitis vinifera: Targeted Metabolomics Analysis as a Tool to Investigate the Chemical Variations in Berries of Four Grapevine Cultivars

Stefania Sut [1],*, Mario Malagoli [2] and Stefano Dall'Acqua [1]

[1] Department of Pharmaceutical and Pharmacological Sciences, University of Padova, Via Marzolo 5, 35121 Padova, Italy

[2] Department of Agronomy, Animals, Food, Natural Resources and Environment, University of Padova, Viale dell'Università 16, 35020 Padova, Italy

* Correspondence: stefania.sut@unipd.it

Table S1. Carbon (C), Nitrogen (N) and Sulfur (S) content in berries. T- berries exposed to 501-treatement; C- control berries. Data are in % of dry weight. *p<0.05 treated versus control

	%C		%N		%S	
	T	C	T	C	T	C
Teroldego	51.05±0.32	51.57±0.56	0.53±0.40	0.51±0.12	0.36±0.13	0.23±0.06
Oseletta	52.8±0.27	51.95±0.97	0.53±0.06	0.50±0.18	0.19±0.07	0.23±0.11
Garganega	51.23±0.73	51.75±0.43	0.37±0.18	0.50±0.16	0.28±0.12	0.29±0.08
Chardonnay	52.40±0.61	52.27±0.66	0.54±0.22	0.43±0.10	0.31±0.18	0.28±0.09

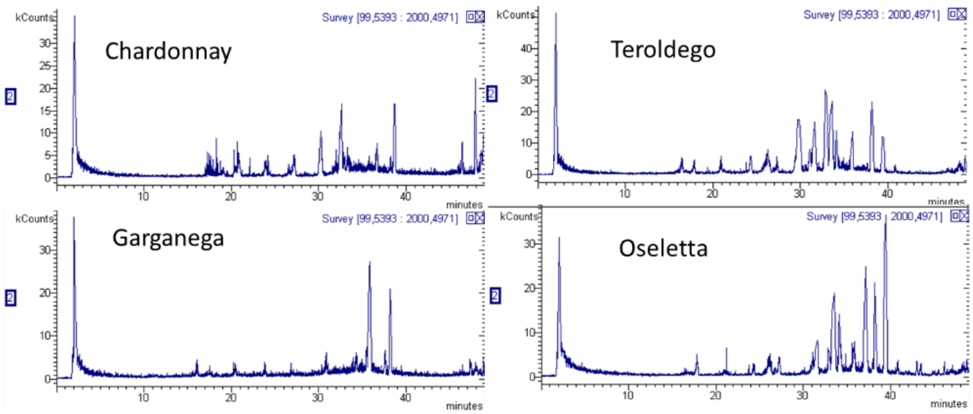

Figure S1. Exemplative LC-MS chromatograms TIC (total ion Current) of the metabolites of the berries in the four grape cultivars.

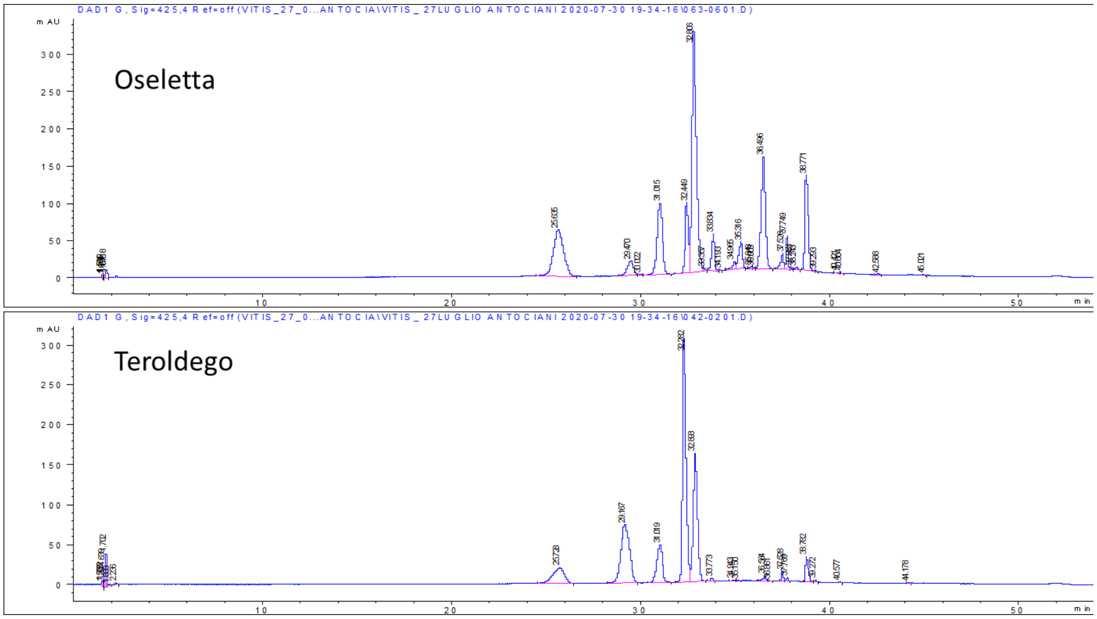

Figure S2. Exemplative enlarged chromatogram at 540nm obtained by LC-DAD of the antocyanins in the berries of Oseletta (above) and Teroldego (below). Peaks were identified on the base of m/z value and fragmentation pathways.

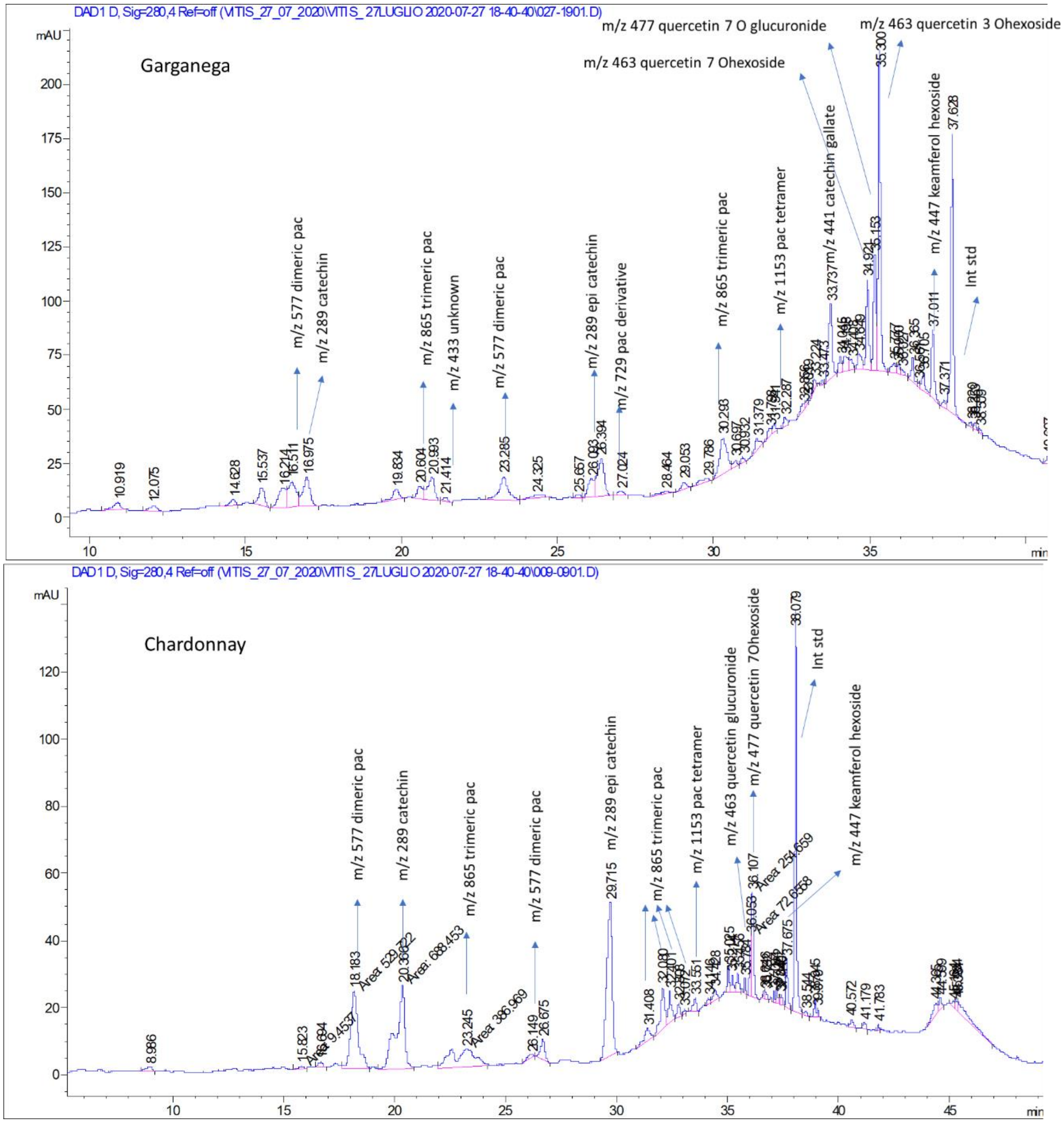

Figure S3. Exemplative chromatogram at 280 nm of Garganega (above) and Chardonnay (below) berries. Peaks were identified on the base of m/z value and fragmentation pathways.

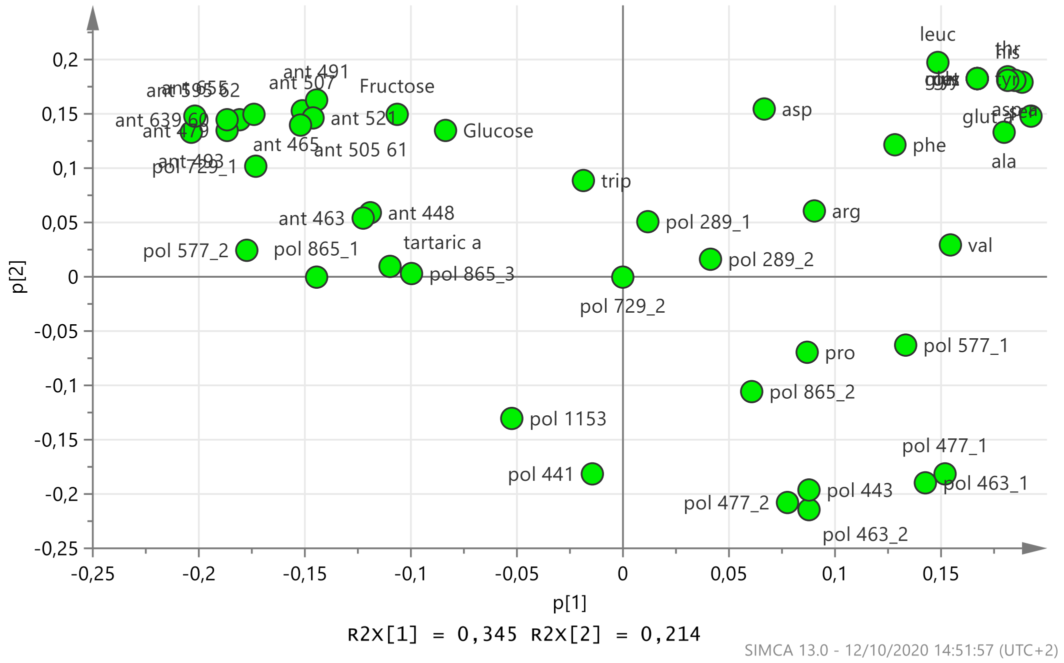

Figure S4. PCA score plot including all the samples of the four cultivars. Each dot represents a variable, namely one metabolite identified and quantified in berry, being a descriptor of the multivariate model.

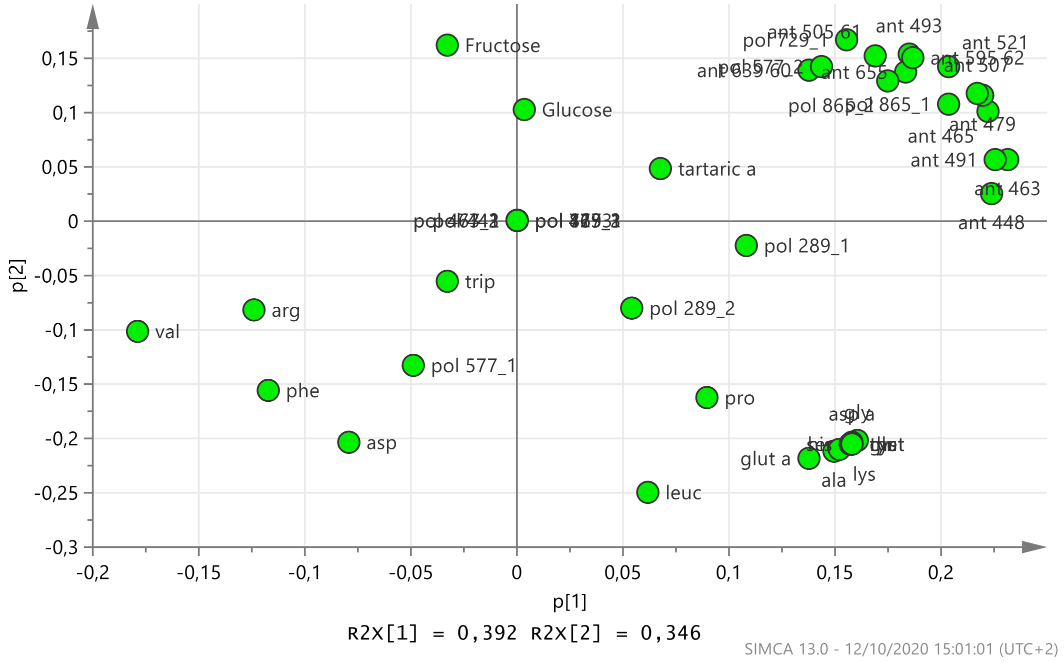

Figure S5. PCA score plot of the Oseletta berries. Each dot represents a variable, namely one metabolite identified and quantified in berry, being a descriptor of the multivariate model.

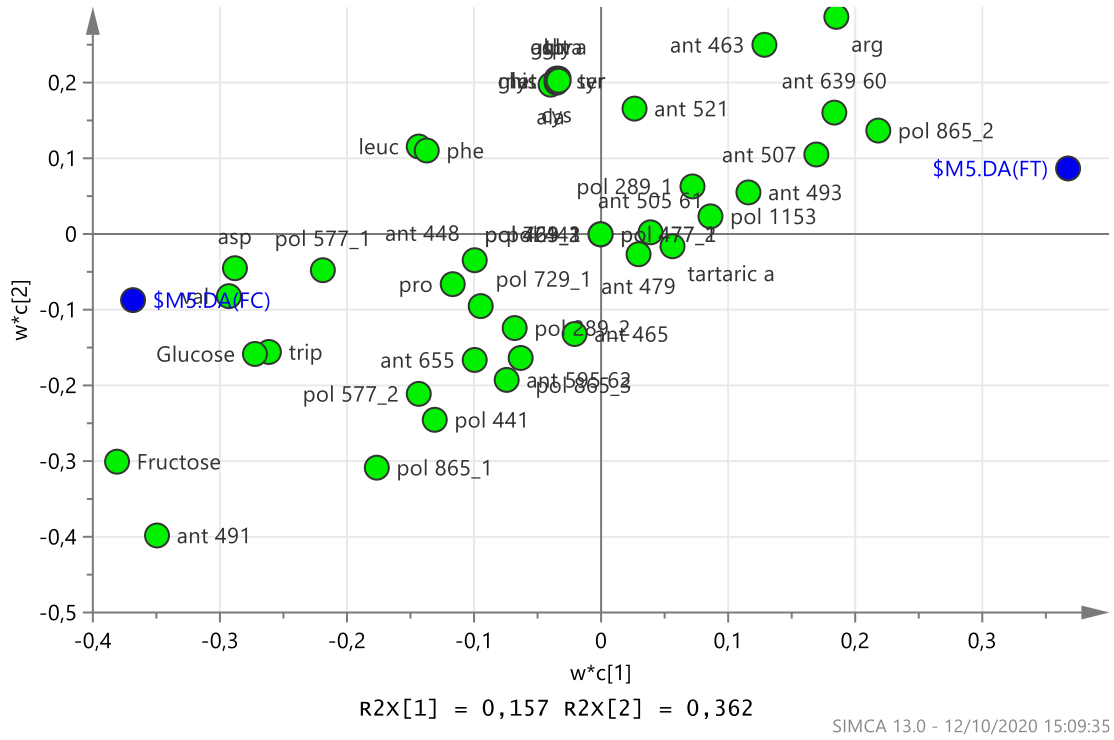

Figure S6. PLS-DA score plot of Teroldego berries. Each dot represents a variable, namely one metabolite identified and quantified in berry, being a descriptor of the multivariate model.

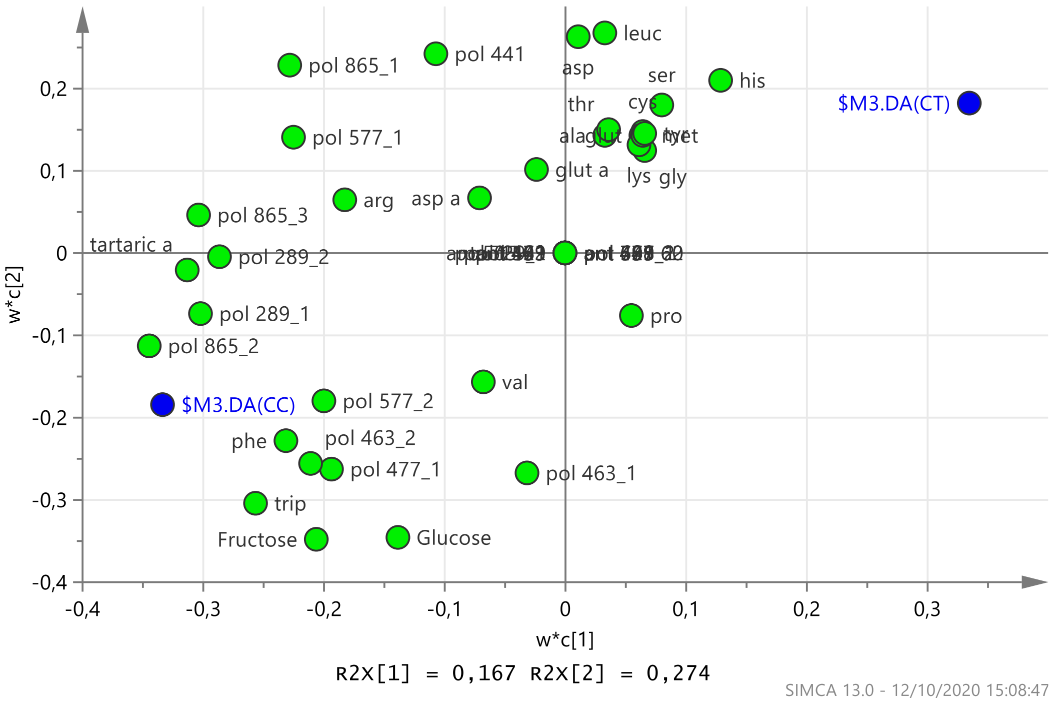

Figure S7. PLS-DA score plot in Chardonnay berries. Each dot represents a variable, namely one metabolite identified and quantified in berry, being a descriptor of the multivariate model.

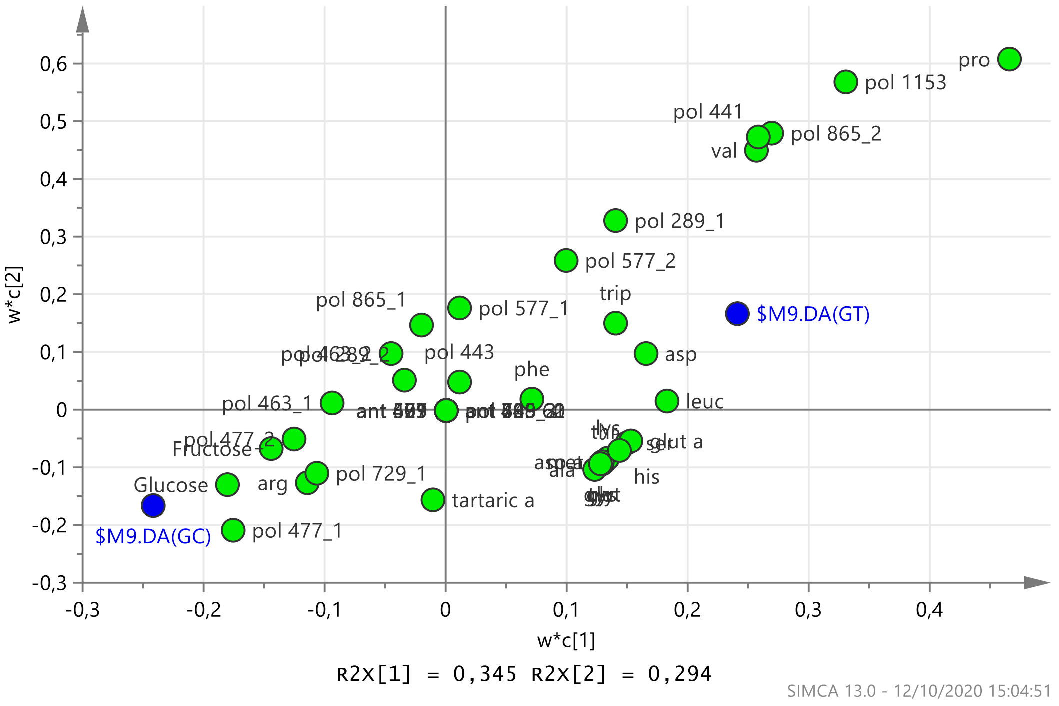

Figure S8. PLS-DA score plot of Garganega berries. Each dot represents a variable, namely one metabolite identified and quantified in berry, being a descriptor of the multivariate model.

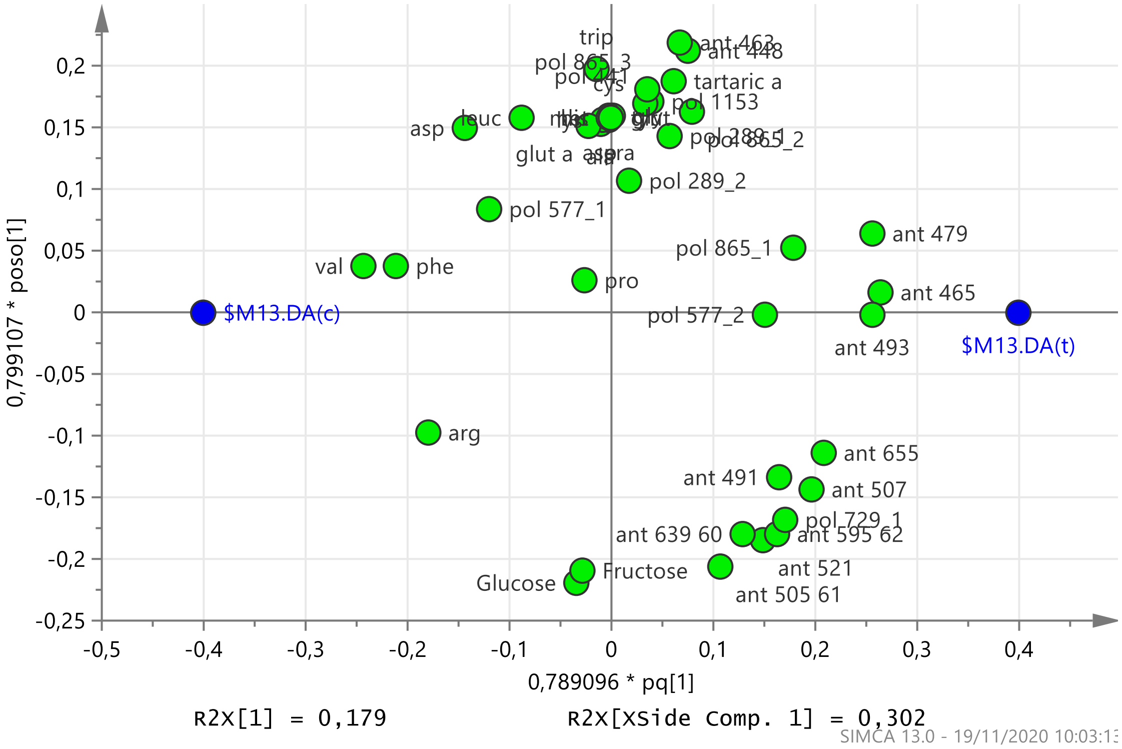

Figure S9. OPLS-DA Score plot of the red grape samples (Oseleta, Teroldego). Each dot represents a variable, namely one metabolite identified and quantified in berry, being a descriptor of the multivariate model

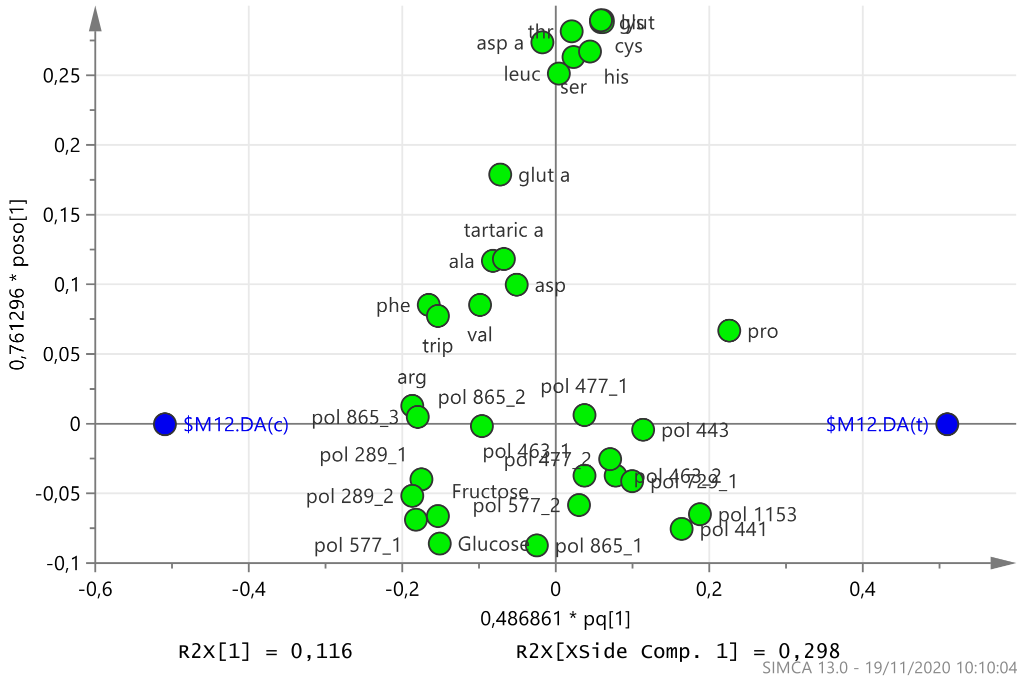

Figure S10. OPLS-DA Score plot of the white grape samples (Garganega, Chardonnay). Each dot represents a variable, namely one metabolite identified and quantified in berry, being a descriptor of the multivariate model.

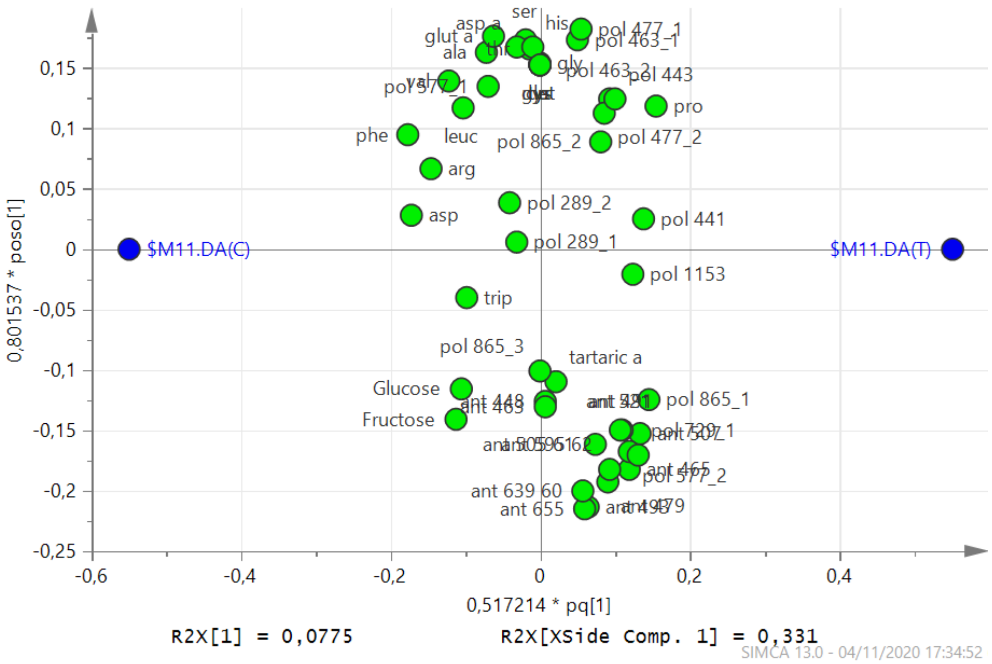

Figure S11. OPLS-DA Loading plot of all the samples (Garganega, Chardonnay, Teroldego, Oseletta). Each dot represents a variable, namely one metabolite identified and quantified in berry, being a descriptor of the multivariate model.

Table S2. abbreviation in multivariate elaboration

compound	Abbreviation
glycine	gly

alanine	ala
serine	ser
proline	pro
Valine	val
Threonine	thr
Cysteine	cys
Leucine	leuc
Asparagine	asp
Aspartic acid	asp a
Glutamine	glut
Lysine	lys
Glutamic acid	glut a
Methionine	met
Histidine	his
Phenylalanine	phe
Arginine	arg
Tyrosine	tyr
Tryptophan	trip
quercetin-7-O-glucoside	pol 463_1
Quercetin-O-glucuronide	pol 477_1
Quercetin-3-O-glucoside	pol 463_2
Kaemferol-O-hexoside	pol 477_2
pac dimer isomer 1	pol 577_1
Catechin	pol 289_1
pac trimer isomer 1	pol 865_1
Unknown	pol 443
pac dimer isomer 2	pol 577_2
Epicatechin	pol 289_2
procyanidin dimer monogallate	pol 729_1
pac trimer isomer 2	pol 865_2
pac tetramer	pol 1153
procyanidin dimer monogallate	pol 729_2
Catechin gallate	pol 441
pac trimer isomer 3	pol 865_3
delphinidin-3-O-glucoside	ant 465
cyanidin-3-O-glucoside	ant 448
petunidin-3-O-glucoside	ant 479
peonidin-3-O-glucoside	ant 463
malvidin-3-O-glucoside	ant 493
delphinidin-3-O-(6''-acetyl-glucoside)	ant 507
cyanidin-3-O-(6''-acetyl-glucoside)	ant 491
petunidin-3-O-(6''-acetyl-glucoside)	ant 521
peonidin-3-O-(6''-acetyl-glucoside) /delphinidin-3-O-(6-p-coumaroyl)glucoside/ malvidin-3-O-(6''-acetyl-glucoside)	ant 505 611 535
malvidin-3-O-(6''-caffeoyl-glucoside)	ant 655
cyanidin-3-O-(6-p-coumaroyl)glucoside/ petunidin-3-O-(6-p-coumaroyl)glucoside	ant 595 625
malvidin-3-O-(6-p-coumaroyl)glucoside / peonidin-3-O-(6-p-coumaroyl)glucoside	ant 639 609

Table S3. Triple quadrupole MS/MS parameters for the analysed amino-acids, polarity, Q1 and Q3 m/z as well as capillary, collision and dwell time are reported.

Compound	Polarity	Q1	Q3	Capillary	Collision	Dwell Time
**Glycine**	Pos.	76.00	30.30	40.000	8.000	0.1
**Alanine**	Pos.	90.00	44.50	40.000	8.000	0.1
**Serine**	Pos.	106.00	60.40	30.000	8.500	0.1
**Proline**	Pos.	116.00	70.30	40.000	10.500	0.1
**Valine**	Pos.	118.00	72.40	30.000	8.500	0.1
**Threonine**	Pos.	120.00	74.40	30.000	8.000	0.1
**Cystine**	Pos.	122.00	75.70	30.000	11.500	0.1
**Isoleucine**	Pos.	132.00	86.50	40.000	7.000	0.1
**Leucine**	Pos.	132.00	86.50	40.000	7.500	0.1
**Asparagine**	Pos.	133.00	87.40	40.000	6.500	0.1
**Aspartic Acid**	Pos.	134.00	74.30	33.876	12.000	0.1
**Glutamine**	Pos.	147.00	84.40	30.000	14.000	0.1
**Lysine**	Pos.	147.10	84.40	30.000	13.500	0.1
**Glutamic Acid**	Pos.	148.00	83.70	30.000	13.000	0.1
**Methionine**	Pos.	150.00	133.40	38.318	6.000	0.1
**Histidine**	Pos.	156.10	110.00	40.000	9.500	0.1
**Phenylalanine**	Pos.	166.10	119.60	40.000	9.500	0.1
**Arginine**	Pos.	175.10	70.40	40.000	18.000	0.1
**Tyrosine**	Pos.	182.10	135.60	40.000	10.000	0.1
**Tryptophan**	Pos.	205.10	187.60	40.000	7.000	0.1
